# Simultaneous diffuse optical and bioluminescence tomography to account for signal attenuation to improve source localization

**DOI:** 10.1364/BOE.401671

**Published:** 2020-10-16

**Authors:** Alexander Bentley, Jonathan E. Rowe, Hamid Dehghani

**Affiliations:** 1 School of Computer Science, College of Engineering and Physical Sciences, University of Birmingham, UK; 2 Physical Sciences for Health Doctoral Training Centre, College of Engineering and Physical Sciences, University of Birmingham, UK

## Abstract

Photonics based pre-clinical imaging is an extensively used technique to allow for the study of biologically relevant activity typically within a small-mouse model. Namely, bioluminescent tomography (BLT) attempts to tomographically reconstruct the 3-dimensional spatial light distribution of luminophores within a small animal given surface light measurements and known underlying optical parameters. Often it is the case where these optical parameters are unknown leading to the use of a ‘best’ guess approach or to direct measurements using either a multi-modal or dedicated system. Using these conventional approaches can lead to both inaccurate results and extending periods of imaging time. This work introduces the development of an algorithm that is used to accurately localize the spatial light distribution from a bioluminescence source within a subject by simultaneously reconstructing both the underlying optical properties and source spatial distribution and intensity from the same set of surface measurements. Through its application in 2- and 3-dimensional, homogeneous and heterogenous numerical models, it is demonstrated that the proposed algorithm is capable of replicating results as compared to ‘gold’ standard where the absolute optical properties are known. Additionally, the algorithm has been applied to experimental data using a tissue mimicking block phantom, recovering a spatial light distribution that has a localization error of ∼1.53 mm, which is better than previously published results without the need of assumptions regarding the underlying optical properties or source distribution.

## 1. Introduction

Pre-clinical photonics based imaging is a powerful non-invasive technique that is used widely to obtain biologically relevant information. An example of this is Bioluminescent Imaging (BLI), where light from distributed biological visible and near-infrared luminophores are detected at the surface of a subject [1]. BLI has been shown to have a variety of useful applications, including detecting and visualizing functional activity within live animals, as well as tracking cells around the body of the animal to uncover potential sanctuary sites such as the brain [2]. The signal measured in BLI is the result of a luciferase-catalyzed reaction which increases within the first minutes, remaining constant for ∼40 minutes [3], therefore giving a safe timeframe for imaging within ∼20 minutes after the luciferin injection [4]. The reactions used in BLI typically provide a low output of bioluminescent photons, however are highly specific leading to very little background signal. Current limitations of BLI include poor spatial resolution, poor depth sampling and low quantitative accuracy which is due to the often low signal intensities, non-linear signal attenuation and the unknown underlying tissue optical parameters.

When requiring to move to a more quantitative measurement, a method can be employed to allow for the recovery of spatially resolved tomographic maps of the source location and intensity, known as bioluminescent tomography (BLT) [5]. BLT utilizes a ‘forward’ model of light propagation within an optically diffuse medium, along with an optimization recovery ‘inversion’ algorithm to accurately reconstruct both the spatial and intensity distribution of the underlying light source. A number of issues arise when attempting to carry out BLT, namely non-uniqueness [6] which is countered through the collection of multi-spectral data. Further issues arise, such as time required to collect multi-spectral data through the use of a filter based system [1] and direct limitations as a result of the effect of bandwidth when using optical filters [7]. These have been previously addressed, through the use of a newly developed hyperspectral imaging system based off compressive sensing, which utilizes a novel method of collecting random projections of an imaging scene using a digital micro-mirror display (DMD) in conjunction with a spectrometer, details of which can be found elsewhere [8].

One of the main limitations that has often been largely ignored is the need for accurate measurements of the underlying optical parameters of the tissue being imaged [9]. Previous work has aimed at accounting for signal attenuation through the use of multi-modal systems that incorporate techniques such as diffuse optical tomography (DOT) to non-invasively obtain the molecular chromophore concentrations as well as spectrally and spatially resolved optical parameters [1]. By using such a system issues arise, mainly through the time required to collect the data through the use of spectral filters along with a high spatially resolved CCD camera. To date a number of strategies both experimental and computational have been employed to improve the accuracy of BLT, including the use of computational models that incorporate permissible regions into the reconstructions. These permissible regions can be defined in multiple ways, for example setting values outside of a specific region to 0 [10], using iterative methods to reduce the area of the permissible region [11], and using structural information gained from other imaging modalities such as MRI [12], CT [13] and ultrasound [14] to improve the reconstruction quality. All of these methods, through either using a multi-modal system or through the use of permissible regions have been shown to significantly improve the accuracy of the results, quoted to be up to 25% in certain cases [14].

Here a novel algorithm is presented, allowing for the simultaneous recovery of chromophore concentrations and optical parameters to aid with accurate localization of the underlying spatial bioluminescence source distribution. This is achieved directly using the same hyperspectral data from the bioluminescence source that can be collected using a hyperspectral imaging system, giving the potential for more accurate localization along with a massive reduction in the time spent collecting data.

## 2. Theory

When developing the methodology and framework for data collection as used in this work, a number of existing algorithms need to be considered. Firstly, a method for obtaining surface fluence data needs to be created. This is done through the use of a compressive sensing (CS) based hyperspectral imaging system as previously described [8], where the premise is to utilize a digital micro-mirror device (DMD) and a spectrometer to collect the spectral data from a sequence of random projections of a scene. By doing this, a set of wavelength dependent linear measurements are built up such that:

(1) $$\begin{array}{c} y_{\lambda_{1}}\\ \vdots \\ y_{\lambda_{i}} \end{array}=\Theta \begin{array}{c} x_{\lambda_{1}}\\ \vdots \\ x_{\lambda_{i}} \end{array},$$

where $y_{\lambda }$ is a M×1 vector of linear compressed measurements at a given wavelength λ, Θ is an M × N matrix that represents the random patterns displayed on the DMD and $x_{\lambda }$. is the original N×1 surface signal that is to be recovered. Taking into account the sparsity present in the signals being measured, it is psible to capture all of the required information to fully represent the original signal with M < N linear measurements [15]. Typically, the correct solution can be found by minimizing the L_1_ norm of $x_{\lambda }$ by constructing the linear convex optimization problem:

(2) $$min\|x_{\lambda }{\|}_{1}\;such\;that\;\Theta x_{\lambda }=y_{\lambda }.$$

This problem can be solved through the use of many different computation algorithms such as primal dual methods [16], Nesterov’s method [17] and conjugant gradient methods [18]. In the case where it is assumed that instead of measuring a sparse signal, the underlying gradient of the image is sparse, which is highly applicable to BLT and BLI, the signal can be recovered by minimizing the total variation (TV) of the signal. This is done by solving a similar linear optimization problem:

(3) $$\min\sum_{i}\|w_{i}\|,\;\;such\;that\;\;\Theta x_{\lambda }=y_{\lambda };\;D_{i}x_{\lambda }=w_{i},$$

where $D_{i}x_{\lambda }$ is the discrete gradient of $x_{\lambda }$ at pixel *i*. TV regularisation has been used since its introduction in 1992 in image denoising [19], image deconvolution [20] and image restoration [21]. Minimizing the TV of a signal is a much more computationally difficult process than standard L_1_ – norm regularisation due to its non-differentiability and non-linearity. One method of working around this is by rewriting the constrained problem as a new sequence of unconstrained sub-problems. This method is used within the total variation minimisation by augmented Lagrangian and alternating direction algorithm (TVAL3) [22–24], which has been utilized in this work and extensively detailed elsewhere [25].

The second problem concerns finding a method of tomographically reconstructing the spatial light distribution from the measured surface fluence data as well as the underlying optical parameters of the subject. Recovering the spatial distribution of the bioluminescence source can be achieved by modelling the problem as a system of linear equations Jb=y, where *J* is a matrix known as the Jacobian, which defines how a small change in source distribution *b*. affects the boundary data measured. Solving this system of linear equations has been shown previously [18] through the implementation of a conjugate gradient approach and is applied in this work through an algorithm known as compressive sensing conjugate gradient (CSCGNW). The conjugant gradient approach solves a number of sub-problems with reducing values of a sparsity weight which converges towards the correct solution, the mathematics and implementation of which have previously been extensively covered [18].

The ultimate algorithm needed to complete the entire framework is one that calculates both the optical properties, given the surface fluence data at multiple wavelengths, as well as the location of the source within the subject. The first (unknown optical properties) is a commonly used imaging technique known as diffuse optical tomography (DOT), named due to the fact that the transport of light through tissue at wavelengths in the visible and near infrared (NIR) bands become near isotropic, therefore is well defined by photon diffusion. A software package, Near infrared fluorescence and spectral tomography (NIRFAST) [26], was developed to allow for the simulation of light propagation within biological tissue using a finite element method (FEM) and is also well documented elsewhere. Within the package are a number of forward and inverse models, however a specific spectrally constrained case is utilised in this work. The algorithm has been modified to take the internal bioluminescence source location and surface fluence data with the goal of directly estimating the concentrations of oxy-hemoglobin, deoxy-hemoglobin and water, as well as scattering power and amplitude [27,28]. The assigned chromophore concentrations can be used to calculate the underlying wavelength dependent absorption coefficient of tissue through the use of extinction coefficients of individual chromophores. This can similarly be done using the scattering power and amplitude to obtain a corresponding reduced scattering coefficient at each wavelengths using Mie scattering theory*.* Using a continuous wave model, the Jacobian is calculated and then inverted using the Moore-Penrose generalised inverse, which is typically more suitable towards underdetermined problems [29]. The Moore-Penrose generalized inverse finds the ‘best fit’ or minimum norm solution to a system of linear equations, the implementation of which have been extensively detailed elsewhere [26,29]. From this an update in optical properties is calculated and the entire process is repeated, new boundary data is simulated and compared with the original data in order to calculate a projection error. This error is then used as a stopping mechanism for when it is considered that convergence has occurred, typically within 2% change. Regularization is used alongside the Moore-Penrose generalized inverse with a starting value of 0.1, which is the standard value used as detailed elsewhere [26].

**Fig. 1.**
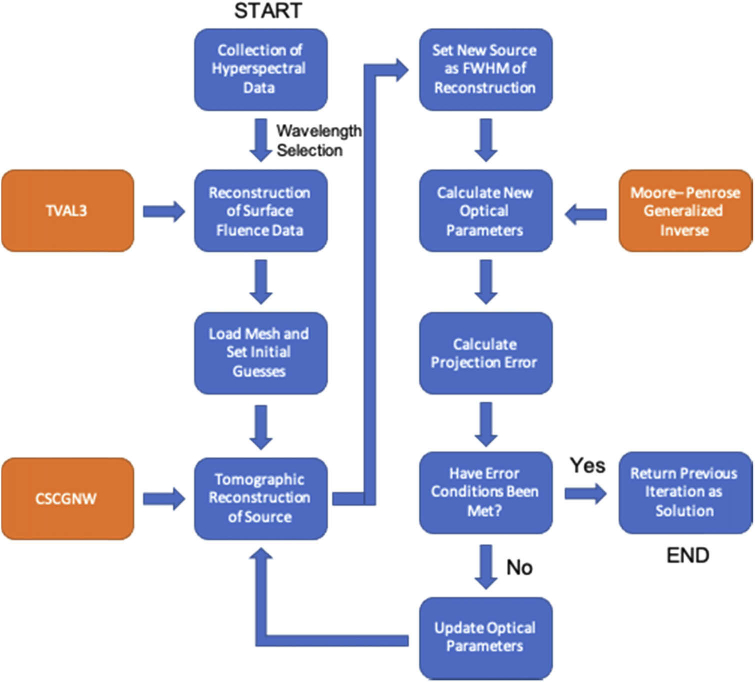
A flowchart of the simultaneous source and optical parameter recovery algorithm. Blue steps represent part of the created algorithm and orange steps represent existing algorithms being used.

Figure 1 is a flowchart showing the sequence of the new iterative algorithm that has been developed by utilising all of the algorithms previously described. Firstly, hyperspectral data is collected using either simulations within NIRFAST or through the collection of real data using a hyperspectral imaging system as defined above. From this data the desired wavelengths are selected and surface fluence data is calculated and combined using the TVAL3 algorithm described above. This data, along with an initial ‘guess’ of underlying optical properties, tomographic reconstruction of the spatial light distribution is achieved using the CSCGNW algorithm. A new source position is set and then through the DOT algorithm, using the same data-set, optical properties of the medium are calculated. From this, if the error conditions have not yet been met, the optical properties are updated by the average value across the entire mesh or of a particular pre-defined volume within*.* Using these updated optical properties, the CSCGNW algorithm is then used again to reconstruct the new spatial light distribution and whole process is completed iteratively. As with the inverse problem outlined above a stopping condition is put in place to stop the algorithm when it is considered to have converged. The stopping criteria is the same as that used in the DOT algorithm whereby a projection error is calculated between the original and modelled data using the updated optical properties. The iterative process is continued until the change in projection error is below a tolerance which is typically set to 2%.

When modelling the new source for the update of optical parameters, two different methods can be used. Firstly the tomographically reconstructed source is represented as its full-width-half-maximum (FWHM) and the center of mass is set as the new point source. Another method is to also represent the reconstructed source as a distributed source, at the recovered FWHM, where the entire distributed source can then be set as the new update. Doing this gives a more realistic representation and therefore may result in more accurate results, both methods are investigated as part of this work.

## 3. Methods and results

### 3.1 System and data collection

**Fig. 2.**
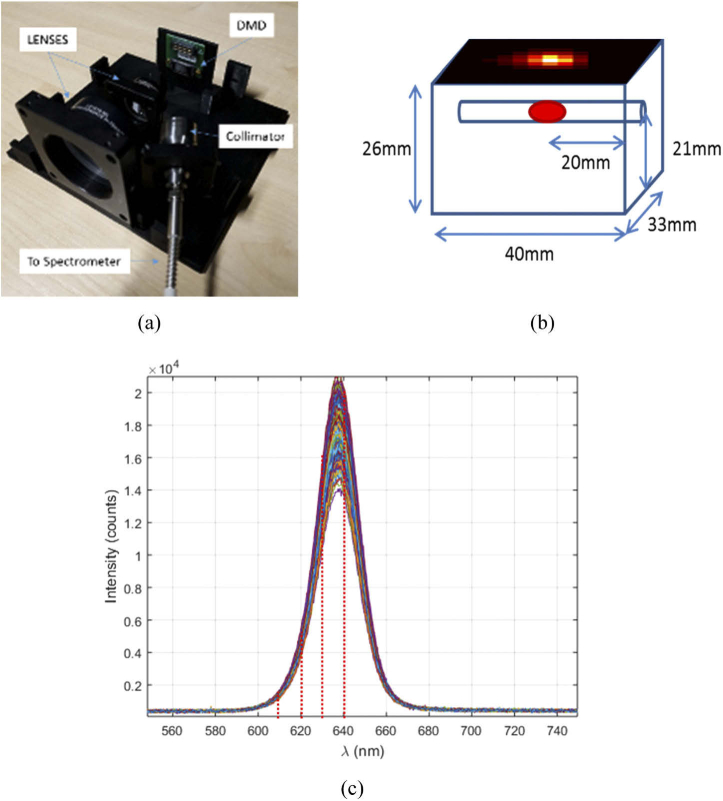
(a) The imaging system developed to collect hyperspectral data from the surface of an object. (b) An example of a tissue mimicking block phantom containing a red light source of ∼ 630 nm, with the surface fluence at 630 nm superimposed on the top surface. (c) The hyperspectral data collect from the surface of a block phantom containing a red LED of ∼ 630 nm. Red dashed line represent the typical selected wavelengths for reconstruction, multicolored solid lines represent an individual measurement taken using the developed system.

Figure 2(a) presents the hyperspectral imaging system that was previously developed [8] to collect measurements from the surface of object of interest. The system contains an optical setup that consists of two lenses, a digital micro-mirror device (DMD) and a collimator. The first lens is a 2 inch diameter concave objective lens which collects light from the imaging plane and focuses it through a 1 inch achromatic lens onto the DMD. A Texas Instruments DLP4500NIR DMD is used, which has a 912 by 1140 array of micro-mirrors that can be individually controlled to be in either an ‘on’ or ‘off’ position. This allows for randomly generated binary patterns taken from a Bernoulli distribution, with probability 0.5, to be displayed, resulting in a random projection of the light from the imaging plane that is incident on the DMD to be directed towards a 1 inch diameter collimator. A Thorlabs F810SMA-635 air-spaced doublet collimator is used to collect this light and pass it through a connected optical fiber of core diameter 1000 µm that is directly connected to a Flame S-VIS-NIR (Ocean Optics) spectrometer. The spectrometer consists of a 200 µm slit and a Sony ILX511B linear silicon CCD array which allows the spectrometer to achieve an optical detection range from 350 nm to 1000 nm with a spectral resolution of 0.4 nm. The entire system and imaging plane are contained in a custom build light-proof housing that ensures elimination of any background light, increasing the signal-to-noise ratio. To control the system a MATLAB graphical user interface has been developed to allow for automated control of both the DMD and spectrometer, once variables such as desired resolution, number of measurements and acquisition time has been defined.

Figure 2(b) represents an example of an object that can be imaged using the system developed, this being a tissue mimicking block phantom containing a red LED which is the subject of Section 3.5. Figure 2(c) represents the hyperspectral data set that is collected by sequentially collecting the spectral data from a set of random projections of the imaging plane using the DMD, where each different colored curve is a separate measurement. From this data, the desired wavelengths and bandwidths (represented by the red dashed lines) are selected and are then fed into the algorithm to first reconstruct the surface fluence of light on the object, an example of which can be seen superimposed on Fig. 2(b). The algorithm then continues to calculate both the optical properties of the object as well as tomographically reconstructing the spatial light distribution located within the object utilizing discrete boundary measurements extracted from the surface. In all cases the reconstructions are based on a threshold of 50% of the maximum value, i.e. the full width half max.

### 3.2 2D numerical experiment

**Fig. 3.**
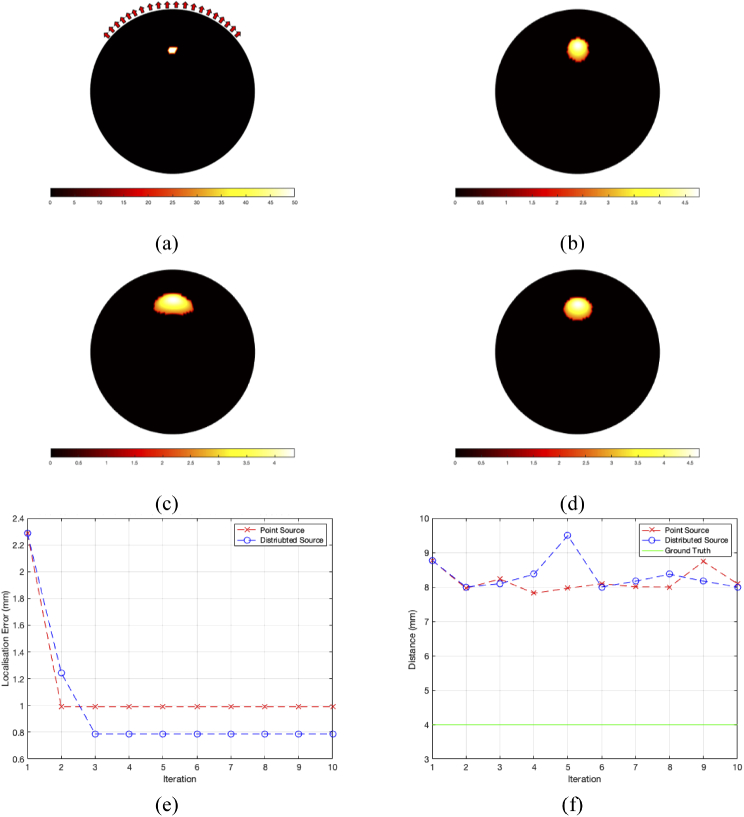
(a) The ground truth location of the light source inside a circle phantom, red arrows represent detector positions. (b) Tomographic reconstruction using the ground truth optical parameters. (c) Tomographic reconstruction of the light source using the initial optical parameter guess. (d) The final tomographic reconstruction of the light source after the error conditions have been met. (e) The localization error between the center of mass of the light source at FWHM and the ground truth location and (f) The FWHM of the recovered source. The red dashed line represents reconstruction carried out with the source modeled as a single point and the blue dashed line represents reconstruction carried out with the whole source at FWHM used.

Reconstructions from simulated data using a homogenous 2D circle model were carried out to initially demonstrate that the algorithm is capable of successfully reconstructing the spatial light distribution located within the model. The circular mesh had a diameter of 50 mm and a source of radius 1.5 mm was placed within the model at a depth of 10 mm from the top surface, as can be seen in Fig. 3(a).

The optical properties and chromophore concentrations of the model were set to that of adipose [26], being 0.012 mM and 0.005 mM for oxy- and deoxyhemoglobin respectively, 40% water and a scattering power and amplitude of 0.56 and 1.34 respectively. Data from the light source was simulated at 4 wavelengths (600 nm, 610 nm, 620 nm and 630 nm) using an array of 20 detectors evenly distributed across the top half of the mesh. Figure 3(b) shows the tomographic reconstruction of the spatial light distribution using the known optical properties and chromophore concentrations of the mesh, representing the gold standard method of reconstruction.

For the simultaneous parameter recovery, the initial guesses of optical properties were set to 0.01 mM for both oxy- and deoxyhemoglobin and 40% for water. As a continuous wave method of calculating the optical properties is currently employed, the scattering parameters are not included in the update and were therefore set to the ground truth values of 0.56 and 1.34 for scattering power and scattering amplitude respectively. Figure 3(c) shows the initial tomographic reconstruction using the set optical parameters, from which the iterative process of calculating the optical parameter updates and source localization begins. Two methods of representing the new source for each optical parameter update were used, the first being a point source located at the center of the reconstruction at FWHM. The second method was representing the new source as the distribution of the reconstruction at FWHM. This iterative process continues until the stopping criteria set out in the previous section have been met, being the second iteration when using a point source and the third iteration when using a distributed source. Figure 3(d) shows the tomographic reconstruction at the third iteration when the source was modelled as the whole distributed source at FWHM. As can be seen in Fig. 3(e) the algorithm performs well in localizing the source, with a localization error of ∼1 mm when using a point source and a better localization error of ∼ 0.8 mm when using the entire distributed source. The localization error is a measure of the Euclidean distance between the reconstructed center of mass (COM) and the known ground truth location, which is defined by:

$$L_{err}=\sqrt{{\left(x_{g}-x_{r}\right)}^{2}+{\left(y_{g}-y_{r}\right)}^{2}+{\left(z_{g}-z_{r}\right)}^{2}},$$

where $x_{g},\;y_{g}\;\text{and}\;z_{g}$ are the Cartesian coordinates of the ground truth location and $x_{r},\;y_{r}\;\text{and}\;z_{r}$ are the Cartesian coordinates of the reconstructed COM. Figure 3(f) shows the calculated FWHM as the maximum distance between two nodes of the reconstructed source, which gives a quantitative measure of the size of the reconstruction. It can be seen that for both methods the recovered sources are ∼ 3 times that of the ground truth, however, this increased size is also found when using the gold standard method of reconstruction for when the underlying optical parameters are known.

### 3.3 3D Homogenous numerical mouse model

**Fig. 4.**
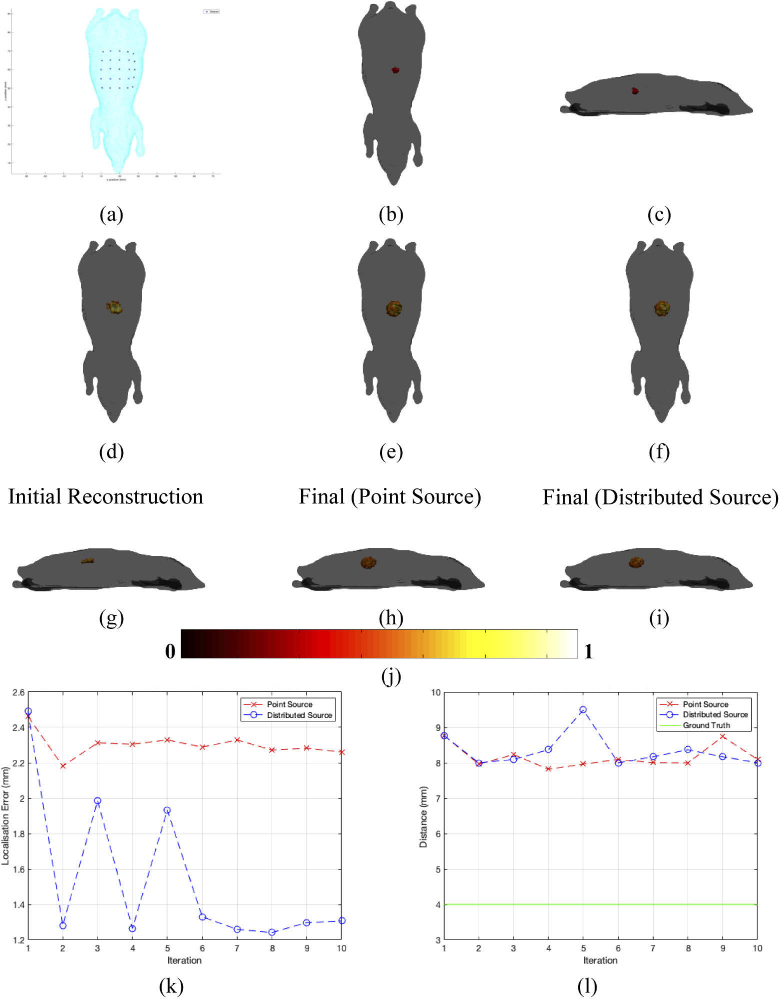
(a) The homogenous mouse phantom used with the detector positions represented by crosses. (b – c) A top and side view of the ground truth light source placed within the phantom. (d – f) The top views of the initial iteration, final iteration using a point source and final iteration using the whole source reconstructions respectively. (g – i) The side views of initial iteration, final iteration using a point source and final iteration using the whole source reconstructions respectively. (j) The colorbar for all reconstructions excluding the ground truth. (k) The localization error between the center of mass of the light source at FWHM and the ground truth location and (l) The FWHM of the recovered source. The red dashed line represents reconstruction carried out with the source modeled as a single point and the blue dashed line represents reconstruction carried out with the whole source at FWHM used. The green line represents the ground truth value.

The second set of simulations carried out were using a homogenous 3D mouse mesh of dimensions ∼ 90 × 40 × 30 mm, which is based on the Digimouse model [30]. The optical parameters of this mesh were set to that of adipose as in the previous example, and a source of diameter 4 mm was placed in the center of the model at a depth of 7.5 mm from the top surface, as shown in Figs. 4(b)–4(c). Surface fluence data from the source was simulated at 4 wavelengths (600 nm, 610 nm, 620 nm and 630 nm) using a 5 × 5 detector array as can be seen in Fig. 4(a). The same process as with the 2D circle example in the previous section was carried out by first reconstructing the spatial light distribution using an initial guess of optical parameters and chromophore concentrations, as shown by the top view in Fig. 4(d) and the side view in Fig. 4(g).

The iterative process was then carried out using both methods of representing the new reconstructed source, as stated in the previous section. Figures 4(e) and 4(h) show the final reconstruction when the source was represented by a point source centered at the reconstruction at FWHM, whilst Figs. 4(f) and 4(i) show the final reconstruction when the source is represented as a distributed source at FWHM. As can be seen in Fig. 4(k), when the new source used for the optical parameter update is represented as a point source the best reconstruction localization error obtained is ∼ 2.2 mm. However, when using the distributed source to obtain the new optical parameters the localization error is much lower at ∼ 1.25 mm. Figure 4(l) shows the calculated FWHM and is show to be ∼ 8 mm for both methods of reconstruction which is ∼ 2 times that of the ground truth of 4 mm.

### 3.4 3D Heterogeneous numerical mouse model

**Fig. 5.**
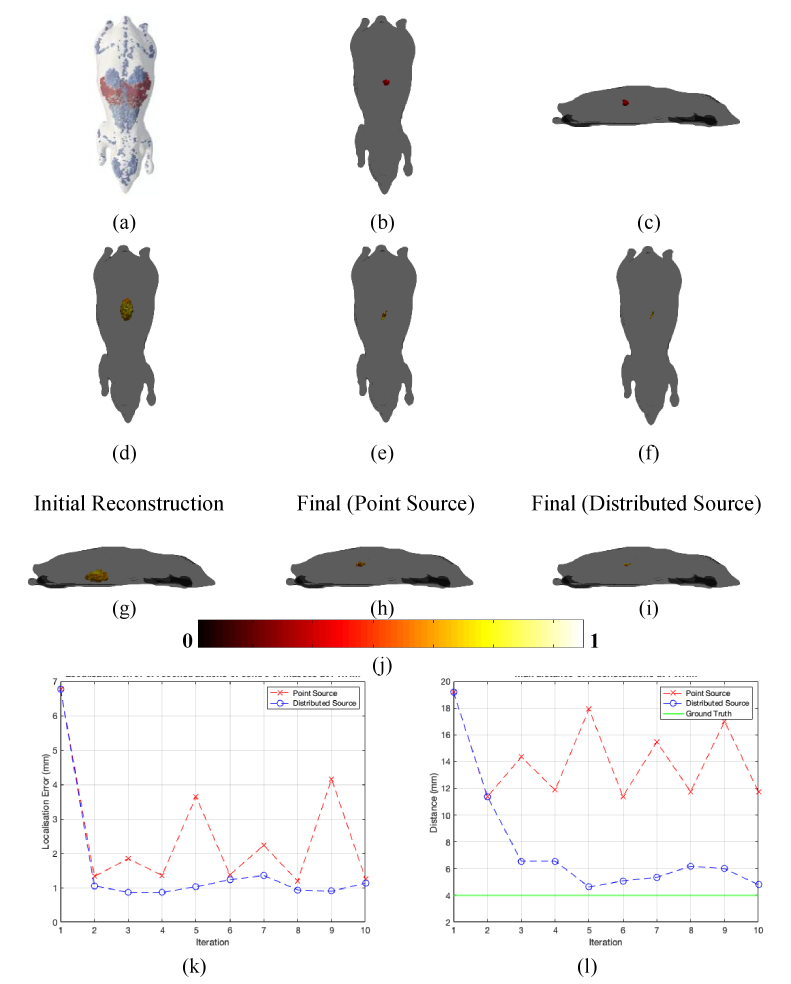
(a) The heterogenous mouse phantom used with regions of varying optical parameters visible. (b – c) A top and side view of the ground truth light source placed within the phantom. (d – f) The top views of the initial iteration, final iteration using a point source and final iteration using the whole source reconstructions respectively. (g – i) The side views of initial iteration, final iteration using a point source and final iteration using the whole source reconstructions respectively. (j) The colorbar for all reconstructions excluding the ground truth. (k) The localization error between the center of mass of the light source at FWHM and the ground truth location and (l) The FWHM of the source. The red dashed line represents reconstruction carried out with the source modeled as a single point and the blue dashed line represents reconstruction carried out with the whole source at FWHM used. The green line represents the ground truth value.

To demonstrate the use of this algorithm on a more realistic model, the same mouse mesh used in the previous section was converted into a heterogenous mesh by marking and setting the optical parameters of 8 separate regions across the mesh to represent different tissue types found in a mouse, Table 1 [31]. Figure 5(a) shows a top down view of the heterogeneous mouse model with the different regions of optical parameter shown. A source of diameter 4 mm was then placed in the center of the model at a depth of 7.5 mm which corresponds to a region that represents the left kidney of the mouse which can be seen in Figs. 5(b)–5(c). Surface fluence data was then simulated at 4 wavelengths (600 nm, 610 nm, 620 nm and 630 nm) using the same 5 × 5 detector array that can be seen in Fig. 4(a).

**Table 1.** The seven different regions that make up the heterogeneous mouse phantom used along with their corresponding chromophore concentrations and scattering properties [31].

Region	Total Hemoglobin (mM)	Oxygen Saturation (%)	Water Concentration (%)	Scatter Amplitude	Scatter Power
Adipose	0.0033	70	50	0.98	0.53
Bone	0.0049	80	15	1.4	1.47
Muscle	0.07	80	50	0.14	2.82
Stomach	0.01	70	80	0.97	0.97
Lung	0.15	85	85	1.7	0.53
Kidney	0.0056	75	80	1.23	1.51
Liver	0.3	75	70	0.45	1.05
Pancreas	0.3	75	70	0.45	1.05

The iterative algorithm was then used using initial optical parameters of 0.00231 mM and 0.00099 mM for oxy- and deoxyhemoglobin respectively as can be seen in Figs. 5(d) and 5(g). The optical parameter updates were calculated using the same two methods of representing the new source as in the previous sections. During each update, instead of updating the global optical parameters as a homogenous model as with the previous sections, spatial a priori knowledge about the different tissues were used. This is achieved by using a priori knowledge of the structure of the mouse to calculate an average of the reconstructed optical parameter for each region. Although such structural data is known in this example, in practice either registration to an atlas may be utilized or structural information from other modalities such as CT [13].

Figures 5(e) and 5(h) show the final tomographic reconstruction of the spatial light distribution when modelling the source used for the optical parameter update as a point source. Figures 5(f) and 5(i) show the final tomographic reconstruction of the spatial light distribution when modelling the source used for the optical parameter update as the whole distributed source at FWHM. It can be seen in Fig. 5(k) that the calculated localization error of the reconstruction is ∼ 1.3 mm when a point source is used, whereas a slightly better localization error of ∼ 0.9 mm when the distributed source is used. The recovered FWHM are displayed in Fig. 5(l) and it can be seen that a maximum of ∼ 11.4 mm is present when a point source is used whereas a maximum of 4.6 mm is found when the whole distributed source is used for reconstruction, which is much closer to the ground truth of 4 mm.

### 3.5 Phantom experimental data

An experiment was carried out to further confirm and demonstrate the capabilities of the proposed algorithm. The imaged object for this experiment was a tissue mimicking block phantom (Biomimic, INQ, Quebec, Canada) of dimensions 33 × 26 × 40 mm. The phantom is made of a solid homogeneous plastic that has spectrally varying optical absorption and scattering properties. These properties have been characterized between the 500 to 850 nm and found to range from, µ_a_ = [0.007–0.12] mm^−1^ and µ_s_´ = [1.63–1.79] mm^−1^ for absorption coefficient and reduced scattering coefficient respectively [32]. The phantom also contains two tunnels with a diameter of 6 mm at depths of 5 mm and 15 mm in which rods of matching or varying optical properties can be inserted to create either a solid homogenous phantom, or a heterogeneous phantom. For the use in this study, a rod of matching optical properties containing an embedded red LED was created to allow it to be inserted into either of the channels to mimic an internal light source, such as a bioluminescent marker. The light source used is a standard 5 mm LED (Arduino) that has a gaussian like emission spectrum with a central peak at ∼ 620 nm and a full-width-half-maximum of ∼20 nm.

**Fig. 6.**
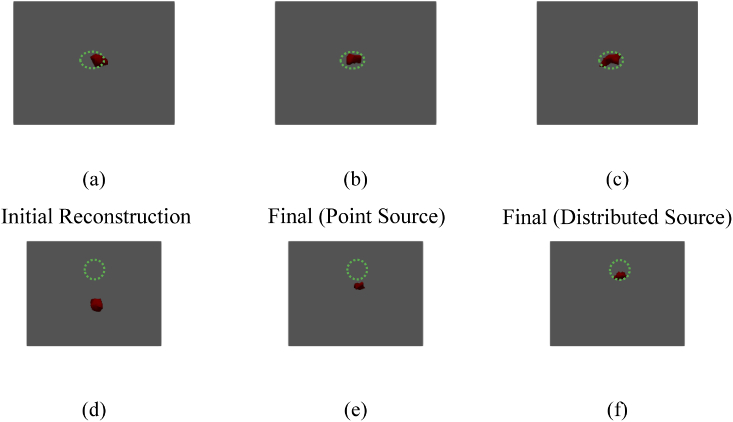
(a – c) Top view of the initial iteration, final iteration modeling the source as a single point and final iteration modeling the whole source respectively. (d – f) Side view of the initial iteration, final iteration modeling the source as a single point and final iteration modeling the whole source respectively. Green dashed area represents the ground truth location of the source. Images are thresholded to 50% of the maximum of recovered value.

For this experiment, the light source was placed within the channel at a depth of 5 mm from the surface. The imaging system shown in Section 3.1 was used to collect a hyperspectral data set. This was done by displaying a sequence of 50 randomly generated 10 × 10 binary patterns, for each pattern collecting spectral data for 200 ms. From this data, surface fluence images were reconstructed using a total variation minimizing algorithm (TVAL3) [22] at 4 wavelengths (610 nm, 620 nm, 630 nm and 640 nm), each with a bandwidth of 10 nm. The initial guesses for chromophore concentrations for this experiment were those of adipose stated in the previous section being 0.00231 mM and 0.00099 mM for oxy- and deoxyhemoglobin respectively. An initial tomographic reconstruction of the spatial light distribution, using the surface fluence data obtained from the system and the initial guesses of chromophore concentrations, can be seen in Figs. 6(a) and 6(d). The iterative algorithm was then used in order to obtain a solution for the location of the light source within the tissue mimicking block phantom, using both a point source and distributed source. Figures 6(b) and 6(e) show the final reconstruction using a point source model and Figs. 6(c) and 6(f) show the final reconstruction using the distributed source model. In all parts of Fig. 6 the ground truth location of the light source within the phantom is represented by the green dashed area.

Table 2 represents the quantitative analysis of the tomographic reconstruction carried out in this experiment. As can be seen using a point source model, the second iteration was returned with a localization error of 3.32 mm and a maximum distance observed at FWHM of 3.91 mm. Compared to a better localization error of 1.53 mm and maximum distance at FWHM of 7.78 mm using a whole distributed source model, which is of the order ∼ 5 times closer to the ground truth size of 7 mm. The chromophore concentrations recovered by the algorithm are 0.0532 mM and 0.0536 mM for oxy- and deoxyhemoglobin respectively. Taking these values and converting them to an underlying absorption coefficient gives a range of µ_a_ = [0.0592–0.1351] mm^−1^ between 610 nm and 640 nm, which represents the ground truth values well.

**Table 2.** Analysis of tomographic reconstructions showing the iteration number returned, the localization error and maximum distance at FWHM for when the sources where modelled as both a point source and a distributed source.

	Iterations	Localization Error (mm)	FWHM (mm)	Ground Truth Size (mm)
Point Source	2	3.32	3.91	7
Distributed Source	6	1.53	7.78	7

## 4. Discussions

Current methods of tomographically reconstructing light sources within an object of interest, for example in bioluminescent tomography, rely on knowing the underlying optical properties of the object in order to obtain an accurate solution. This can be done through either a priori information for example from ATLAS based information [33,34] or through the use of multi modal systems that have the capability of directly measuring such properties [1]. The work in this paper outlines the development and testing of an algorithm that uses fluence data from an internal light source at the surface of the object of interest to reconstruct both the source localization and underlying optical properties of the object simultaneously. Taking advantage of a previously developed system [8], that is capable of collecting hyperspectral surface fluence data, a number of simulation and real data experiments were designed in order to test the ability of the algorithm in finding a correct solution to the problem. By doing this a vast amount of time is saved in data collection as compared to existing methods as they typically use a manually changed spectral filter approach in order to build up a multi-spectral dataset.

The algorithm developed has shown to perform well in localizing the spatial light distribution present in both 2 and 3-dimensional simulated cases, as well as homogenous and heterogeneous mouse phantoms. Real experimental data was then tested to see if the outcomes found in simulations could be replicated in an experimental setting. This was done by collecting hyperspectral surface fluence data using the system described in Section 3.1 and applying the algorithm presented to the dataset. To further test the robustness of the algorithm when optical parameter updates were being calculated, the source location used was modeled as a point source centered at the reconstructed light distribution at full-width-half-maximum, as well as modelling the source as the entire spatially distributed light source reconstructed at FWHM. It was found in all cases that the algorithm performed well at localizing the source distribution as well as obtaining good volumetric accuracy, especially in the more realistic examples demonstrated in Sections 3.4 and 3.5.

In order to quantitatively analyze the accuracy of reconstruction, two different metrics were used. The first being the localization error, which is a measure of the Euclidean distance between the center of mass of the reconstructed distribution at FWHM and the ground truth location. The second metric use was the maximum Euclidean distance between nodes present within the distribution at FWHM, which gives a direct indication of the size of the reconstruction. In the 2D case, good localization was seen with errors of ∼ 1 mm and 0.8 mm for a point source model and distributed model respectively. A maximum FWHM of 10 mm was found when using both models, which is ∼ 3 times that of the ground truth, however this increase is also seen when reconstructing the source using the known ground truth optical parameters. When utilizing simulations of a 3-dimensional mouse phantom, similar results were seen, with localization errors of ∼ 2.2 mm and 1.25 mm for when a point source model and distributed source model was used respectively. The calculated FWHM was found to be ∼ 8 mm for both models, which is ∼ 2 times that of the ground truth. A more realistic example was explored through the use of a heterogeneous mouse model containing 8 distinct regions of different optical properties that relate directly to the anatomy of a real mouse. When using a point source model, a localization error of 1.3 mm was found along with a FWHM of 11.4 mm, which is much larger than the ground truth value of 4 mm. However, when modeling the update source as the whole distributed reconstruction, much better accuracy was gained with a localization error of 0.9 mm and a FWHM of 4.6 mm. These results were mirrored when using real experimental data, with a localization error of 3.32 mm and maximum distance of 3.91 mm when a point source model was used. As compared to when a whole distributed source model was used a localization error of 1.53 mm and a FWHM of 7.78 mm was calculated, being ∼ 5 times closer to the ground truth value of 7 mm that in the point source model case. When compared to similar data [8], it can be seen that by using this algorithm for reconstruction, results with much better accuracy can be observed.

This proposed algorithm is aiming to account for the underlying unknown optical parameters in order to gain a tomographic reconstruction with improved accuracy and quality. The optical absorption coefficient recovered for the experiment phantom ranged between µ_a_ = [0.0592–0.1351] mm^−1^ for the wavelengths used. Although these values match well with the ground truth values of µ_a_ = [0.007–0.12] mm^−1^, further work is required to look at its biological relevance. When carrying out tomographic reconstructions using a heterogeneous mouse model (or a real murine example), the use of a priori knowledge of the structure of the mouse has been utilized. This allows for the algorithm to account for small areas of high absorption within the mouse, without the need for directly measuring the optical properties of these regions This information can be gained using methods such as the permissible regions techniques explained above or through the use a mouse atlas such as Digimouse [30], to infer the internal structure of the mouse from measurements of key features on the surface of the mouse.

Using this new algorithm to tomographically reconstruct spatially distributed internal light sources, by simultaneously recovering the underlying optical properties and localizing the source can potentially address a number of issues that typically arise in tomographic imaging, such as bioluminescent tomography (BLT). The first of which is accounting for the unknown optical parameters of the subject, which is typically met by directly measuring the optical parameters using a multi-modal system [1], resulting in a vast increase in data collection time, which is especially valuable in cases such as BLT where the timeframe of light emission is finite. The system developed allows for the collection of hyperspectral data without the need of spectral filters to be used, further giving the potential for the data collection time to be reduced. The use of a filter-less system also addresses issues that are raised in filter based systems, whereby the bandwidth of said filters have an effect on measured data [7]. The effective bandwidth and number of spectral measurements made are limited by only the spectral resolution of the spectrometer used, therefore can be controlled well and is a topic for future work.

## 5. Conclusions

This work highlights the development of an algorithm to be used in conjunction with a previously presented hyperspectral imaging system [8]. The main aim of the algorithm is to achieve better source localization by simultaneously calculating and updating the underlying optical properties and tomographically reconstructing the spatial light distribution using an iterative method. The algorithm has shown to give solutions with good localization accuracy (∼1 mm) with both 2 - and 3 – dimensional simulations using homogenous, heterogenous models as well as real experimental data from a tissue mimicking block phantom. Good volumetric accuracy was also achieved in the heterogenous mouse model and experimental data when modeling the updated source as the whole distributed reconstruction at FWHM. These results have the potential to directly translate to improvements in data collection times, especially in multi-modal multi-spectral systems where the underlying optical properties are unknown. It is believed that the algorithms accuracy and efficiency could be improved further through the use of spectral derivative data, as this has been shown previously to improve the accuracy of tomographic reconstruction of bioluminescent light sources [33]. As hyperspectral data is collected with the system presented, it is possible that the system and algorithm could be utilized to simultaneously collect data from multiple sources of different wavelengths. Other possible improvements could be gained from the true utilization of the hyperspectral data by incorporating a much greater number of wavelengths at each stage of the algorithm and optimizing the bandwidth of data used all of which will be subject of further investigation.
